# Age-period-cohort analysis of kidney cancer deaths attributable to high body-mass index in China and U.S. adults

**DOI:** 10.1186/s12889-020-09007-7

**Published:** 2020-06-08

**Authors:** Xiaoxue Liu, Yong Yu, Minsheng Wang, Fang Wang, Sumaira Mubarik, Yafeng Wang, Runtang Meng, Chuanhua Yu

**Affiliations:** 1 Department of Epidemiology and Biostatistics, School of Health Sciences, Wuhan University, No. 115, Donghu Road, Wuhan, 430071 China; 2 School of Public Health and Management, Hubei University of Medicine, Shiyan, 442000 China; 3 The First Affiliated Hospital of Anhui Medical University, Anhui, 230022 China; 4 Global Health Institute, Wuhan University, Wuhan, 430072 China

**Keywords:** Kidney cancer, Mortality, High BMI, Age-period-cohort effect, Trend

## Abstract

**Background:**

Statistical data on burden of kidney cancer and the relavant risk factors are valuable for policy-making. This study aims to estimate kidney cancer deaths and high body-mass index (BMI) attributable to the deaths by gender and age group in China adults, compared with U.S.

**Methods:**

We extracted kidney cancer data (1990–2017) about the age-standardized rates using the comparative risk assessment framework of the 2017 Global Burden of Disease study. We performed an age-period-cohort (APC) analysis to estimate trends of kidney cancer mortality attributable to high BMI.

**Results:**

During 1990–2017, age-standardized mortality rate of kidney cancer was increasing in China but decreasing in U.S. The mortality attributable to high BMI in China showed a general increasing trend, while that in U.S. men was increasing and tended to be stable in women since 1995. APC analysis showed a similar pattern of age effect between China and U.S. adults, which substantially increased from 20 to 24 to 90–94 age group. Differently, the period effect rapidly increased in China than U.S. adults during 1990–2017. The cohort effect peaked in the earlier cohort born in 1902–1906 in China, and it declined consistently in U.S. with exception of 1902–1906 and 1907–1911 birth cohort.

**Conclusions:**

The kidney cancer deaths attributable to high BMI, and period effect have been generally increasing in China adults, compared with U.S. adults in which the trend tends to be stable in recent years. The rapid aging may also intensify the increasing trend of kidney cancer death in China. Effective measures should be conducted on body weight control and care for kidney cancer prevention.

## Background

Kidney cancer has been the common cancers in the world [1, 2]. There are 48,210 new cases and 17,168 deaths in China in 2017, accounting for 12.27% and 12.39% of 393,042 new cases and 138,528 deaths worldwide, respectively [2]. Kidney cancer mainly includes renal cell carcinoma with a proportion of 90–95% [3]. The incidence of kidney cancer has increased year by year in recent years, rising by 2% to 3% compared with 10 years ago [4]. The mortality of kidney cancer has been stable globally since the 1990s, while it decreases in most countries in recent years [5]. In China, both incidence and mortality of kidney cancer seemed low, but previous study reported incidence of kidney cancer had greatly increased [6]. The mortality trend of kidney cancer remains unknown.

Established risk factors for kidney cancer mainly include tobacco smoking, obesity/overweight, hypertension and chronic kidney disease [5, 7–10]. It is currently accepted that there is a global epidemic of obesity [11]. The prevalence of overweight or obesity in China adults is also rising greatly during 1993–2014 [12, 13]. It is being concerned that obesity increased the risk of kidney cancer [8, 14]. This relationship is attributed to abnormal secretion of adipokines, insulin resistance, higher estrogen level among overweight/obesity individuals [9, 10]. However, with a rapid urbanization and transition to western dietary and lifestyle in China, people experienced an increasing exposure to obesity [6, 15], and trends of kidney cancer death remains unknown. The prevalence of obesity/overweight is different between China and U.S. populations. Therefore, we aim to further estimate the time patterns of kidney cancer mortality attributable to high BMI to present the relative risk due to age, period and cohort of the mortality in China, compared with U.S., which could provide epidemiological evidence for kidney cancer prevention and control.

## Methods

### Data sources

This study obtained kidney cancer data from global burden of disease (GBD) 2017 study. GBD 2017 study provided a comprehensive estimation of annual incidence, prevalence, mortality for causes of death and the corresponding risk factors in 195 countries and territories during 1990–2017 [16, 17]. The GBD study summaries burden of disease for global populations among different causes, locations, ages, and sexes [18]. The authors declare all the data used in the study was deidentified. Ethics was not required because the data was publically available.

The age-standardized mortality rates (ASMR) of kidney cancer attributable to high BMI were obtained from GBD 2017 [19]. The GBD study attributes each death to a single underlying cause that began the series of events leading to death, in accordance with ICD principles. Kidney cancer was defined as the International Classification of Diseases of the 10th revision (ICD-10) code C64 and code 189 in ICD-9 [16]. The original mortality database is composed of vital registration (VR), verbal autopsy (VA), registry, survey, police, and surveillance data [1, 20]. The GBD study organizes causes of death in a hierarchical list (four levels), which was further refined to separately estimate causes with substantial policy interest or high levels of burden. To predict the level for each cause of death, GBD 2017 used the Cause of Death Ensemble model (CODEm) to systematically test a large number of functional forms and permutations of covariates [16]. The original data of kidney cancer mortality in China population was mainly from the Cause of Death Reporting System of the Chinese Center for Disease Control and Prevention (CDC), Disease Surveillance Points (DSPs) and the Maternal and Child Surveillance System, which are considered to be nationally representative [21]. Mortality data sources of both China and U.S were classified into cancer groups according to the International Classification of Diseases and estimated by GBD study. To increase the comparability of the two regions, age-standardized mortality rates were calculated adjusted to the GBD 2017 global standard population using the direct method. In this analysis, mortality data of the population aged under 20 years old was excluded for both China and U.S. because kidney cancer diagnosed in children is rare. The mortality of people aged above 95 years old was also excluded because these data couldn’t meet APC analysis.

### Attributable burden

The GBD study incorporated the comparative risk assessment framework previously to quantify the burden of several causes and impairments attributable to 84 environmental, occupational, metabolic, and behavioral risk factors. Systematic literature search was performed in PubMed to find the evidence for kidney cancer deaths due to the attributable risk factors. For each included study, the proportions of kidney cancer cases induced by the specific risk factors were calculated with exception of outliers or data that did not meet the inclusion criteria [2]. Briefly, after assessing the casual evidence in each risk-outcome pair, we analyzed the attributable burden of kidney cancer deaths to high BMI. Overweight and obesity are defined by measures of weight and height that provide an index of one’s mass, referred to as a BMI. In this study, based on comparative risk assessment from GBD 2017 study, high body-mass index (BMI) for adults (ages 20+) is defined as BMI greater than theoretical minimum risk exposure level: 20–25 kg/m^2^ [22].

### Statistical analysis

In the descriptive analysis, the age-standardized mortality rate (per 100,000 population) was calculated according to the direct method by summing up the products of age-specific rates (*α*_*i*_, where *i* denotes the *i* th age class) and the number of persons (*β*_*i*_) in the same age subgroup *i* of the chosen reference standard population, followed by dividing the sum of the standard population weights, i.e., [23].

$$ ASMR=\frac{\sum_{i=1}^A{\alpha}_i{\beta}_i}{\sum_{i=1}^A{\beta}_i}\times 100,000 $$

Standardization was considered imperative for this study as it eliminates the bias when comparing the rates between the two areas.

The APC model could be expressed as:

$$ {Y}_J=\mu +a\kern0.5em {age}_j+\beta {period}_j+\gamma {cohort}_j+{\varepsilon}_i $$

where *Y*_*J*_ denoted the response variable—the net effect on colorectal cancer mortality for group *j*, *a*, *β* and *γ* denoted the coefficient of age, period and cohort of APC model, respectively, and *μ* denoted the intercept of the model. *ε*_*i*_ denoted the residual of the APC model.

In this model, the cohort can be expressed by age and period; that is, cohort = period−age. An on-identification problem may still exist as there is a linear relationship between the age, period and cohort. In our study, APC model with an intrinsic estimator (IE) method was used to solve the collinearity problem, which is based on estimable functions and the singular value decomposition of matrices [24].

In this analysis, age reflects variations in vital rates, based on that mortality risk increases with the process of ageing. Period effects represent influential factors, including complex sets of historical events and environmental factors, that simultaneously affect all age groups. Cohort effects represent variations across groups of individuals born in the same year or years. The age, period and cohort effects influence morbidity and mortality risks of disease in specific ways, especially period effect represent complex sets of historical events and environmental factors [1]. APC analysis was used to decompose the three trends in mortality and provides unbiased and relatively efficient estimation results [25]. The conventional approaches include two-factor models (age-period (AP), age-cohort (AC), and period-cohort (PC) models) and constrained generalized linear models (CGLIMs). APC model was commonly selected for estimating age, period and cohort effect of disease data [24, 26]. Goodness-of-fit statistics and the best-fitting model was presented in Supplementary Table 1.

In this work, age-specific rates of kidney cancer mortality attributable to high BMI were classified by consecutive age groups (20–24, 25–29, ..., 90–94), 5-year periods (1992, 1997, 2002, 2007, 2012, 2017), and correspondingly 5-year birth cohort groups (1902–1906, 1907–1911, …, 1997–2001). The estimated coefficients for the age, period and cohort effects by APC analysis was shown in Supplementary Table 2A and 2B, and then these coefficients were calculated to the exponential value (exp(coef.) = e^coef.^) which denotes the mortality relative risk (RR) of a particular age, period, or birth cohort relative to each average level (see Tables 1 and 2). For example, for period effect of China men, the risk of kidney cancer due to high BMI in 2017 was 6.23 (exp. (*β*_2017_ - *β*_mean_) − (*β*_1992_ − *β*_mean_) = exp. (*β*_2017_ - *β*_1992_) ≈ 6.23) times the risk in 1992. This analysis was performed by Stata 14.0 software (StataCorp, College Station, TX, USA).

**Table 1:** The relative risks of kidney cancer mortality attributable to high BMI due to age, period and cohort effects, China

Factor	Men			Women		
	RR	95% CI		RR	95% CI	
		Lower	Upper		Lower	Upper
**Age**						
20–24	0.07	0.00	4.93 × 10^5^	0.11	0.00	2.52 × 10^5^
25–29	0.13	0.00	2322.96	0.17	0.00	3853.25
30–34	0.21	0.00	410.40	0.20	0.00	1275.74
35–39	0.31	0.00	168.34	0.25	0.00	478.42
40–44	0.50	0.00	89.29	0.40	0.00	159.30
45–49	0.82	0.01	60.57	0.67	0.01	83.08
50–54	1.22	0.03	47.55	0.98	0.02	57.00
55–59	1.62	0.07	36.81	1.40	0.05	41.47
60–64	1.91	0.14	26.61	2.03	0.13	31.29
65–69	2.23	0.25	19.89	2.74	0.30	25.00
70–74	2.63	0.42	16.29	3.29	0.51	21.10
75–79	3.50	0.73	16.75	3.52	0.62	19.90
80–84	3.57	0.71	17.98	3.34	0.51	21.85
85–89	4.07	0.63	26.21	3.96	0.45	34.84
90–94	3.96	0.40	39.22	3.63	0.25	52.85
**Period**						
1992	0.39	0.05	2.85	0.49	0.06	3.99
1997	0.49	0.11	2.16	0.59	0.12	2.80
2002	0.82	0.28	2.38	0.85	0.27	2.70
2007	1.31	0.50	3.40	1.18	0.40	3.44
2012	1.98	0.61	6.47	1.68	0.45	6.22
2017	2.44	0.50	11.88	2.05	0.36	11.79
**Cohort**						
1902–1906	3.29	0.04	276.04	2.70	0.02	355.62
1907–1911	2.68	0.08	91.34	2.40	0.05	110.30
1912–1916	2.27	0.12	41.76	2.15	0.09	50.90
1917–1921	1.97	0.17	22.35	1.90	0.13	27.13
1922–1926	1.89	0.24	15.10	1.80	0.18	17.56
1927–1931	1.76	0.26	11.84	1.74	0.22	13.98
1932–1936	1.55	0.20	12.23	1.64	0.17	15.64
1937–1941	1.35	0.12	14.80	1.52	0.11	20.67
1942–1946	1.11	0.07	18.81	1.37	0.06	28.99
1947–1951	1.01	0.04	28.23	1.27	0.04	45.26
1952–1956	1.04	0.02	45.92	1.26	0.02	78.05
1957–1961	0.92	0.01	69.02	1.06	0.01	127.64
1962–1966	0.78	0.01	103.57	0.82	0.00	216.10
1967–1971	0.78	0.00	170.81	0.78	0.00	387.01
1972–1976	0.64	0.00	295.45	0.63	0.00	787.25
1977–1981	0.50	0.00	769.12	0.48	0.00	3280.14
1982–1986	0.45	0.00	2792.20	0.42	0.00	17,949.19
1987–1991	0.42	0.00	21,024.40	0.38	0.00	1.23 × 10^5^
1992–1996	0.37	0.00	2.15 × 10^6^	0.32	0.00	6.76 × 10^6^
1997–2001	0.26	0.00	1.36 × 10^14^	0.21	0.00	4.28 × 10^14^

Notes: *RR* Relative risk [RR = exp.(coefficient)], *CI* Confidence interval

**Table 2:** The relative risks of kidney cancer mortality attributable to high BMI due to age, period and cohort effects, U.S.

Factor	Men			Women		
	RR	95% CI		RR	95% CI	
		Lower	Upper		Lower	Upper
**Age**						
20–24	0.05	0.00	13.34	0.08	0.00	17.08
25–29	0.07	0.00	4.30	0.11	0.00	6.18
30–34	0.12	0.01	2.80	0.14	0.00	4.35
35–39	0.25	0.02	2.48	0.24	0.02	3.41
40–44	0.54	0.09	3.08	0.43	0.05	3.42
45–49	1.06	0.26	4.25	0.76	0.15	3.97
50–54	1.70	0.54	5.36	1.24	0.32	4.73
55–59	2.38	0.93	6.06	1.79	0.60	5.32
60–64	2.88	1.35	6.13	2.37	0.99	5.71
65–69	3.18	1.73	5.85	2.89	1.43	5.85
70–74	3.31	1.96	5.59	3.35	1.84	6.09
75–79	3.38	2.00	5.70	3.55	1.96	6.42
80–84	3.07	1.66	5.68	3.73	1.88	7.38
85–89	3.49	1.65	7.38	4.13	1.79	9.54
90–94	3.50	1.38	8.84	4.18	1.48	11.77
**Period**						
1992	0.62	0.34	1.14	0.70	0.36	1.37
1997	0.76	0.51	1.14	0.82	0.52	1.29
2002	0.94	0.72	1.21	0.95	0.70	1.29
2007	1.09	0.84	1.42	1.07	0.79	1.46
2012	1.30	0.88	1.93	1.21	0.77	1.90
2017	1.58	0.90	2.78	1.43	0.75	2.71
**Cohort**						
1902–1906	2.31	0.54	9.95	2.31	0.47	11.21
1907–1911	2.45	0.76	7.91	2.44	0.69	8.65
1912–1916	2.56	0.97	6.79	2.52	0.89	7.19
1917–1921	2.52	1.11	5.71	2.51	1.05	6.02
1922–1926	2.42	1.19	4.95	2.39	1.12	5.10
1927–1931	2.21	1.13	4.31	2.17	1.06	4.42
1932–1936	1.97	0.96	4.01	1.93	0.89	4.19
1937–1941	1.72	0.76	3.90	1.70	0.69	4.21
1942–1946	1.49	0.57	3.90	1.46	0.50	4.29
1947–1951	1.26	0.41	3.89	1.22	0.34	4.40
1952–1956	1.03	0.28	3.85	1.00	0.22	4.53
1957–1961	0.86	0.19	3.93	0.85	0.15	4.90
1962–1966	0.70	0.12	4.02	0.72	0.10	5.40
1967–1971	0.56	0.08	4.17	0.57	0.06	5.99
1972–1976	0.46	0.04	4.74	0.46	0.03	7.37
1977–1981	0.40	0.02	7.14	0.41	0.01	12.41
1982–1986	0.38	0.01	15.64	0.40	0.01	26.98
1987–1991	0.36	0.00	52.63	0.37	0.00	83.92
1992–1996	0.33	0.00	363.70	0.33	0.00	483.03
1997–2001	0.29	0.00	90,958.62	0.28	0.00	1.33 × 10^5^

**Notes:***RR* Relative risk [RR = exp.(coefficient)], *CI* Confidence interval

## Results

### Age-standardized mortality rates of kidney cancer

**Fig. 1:**
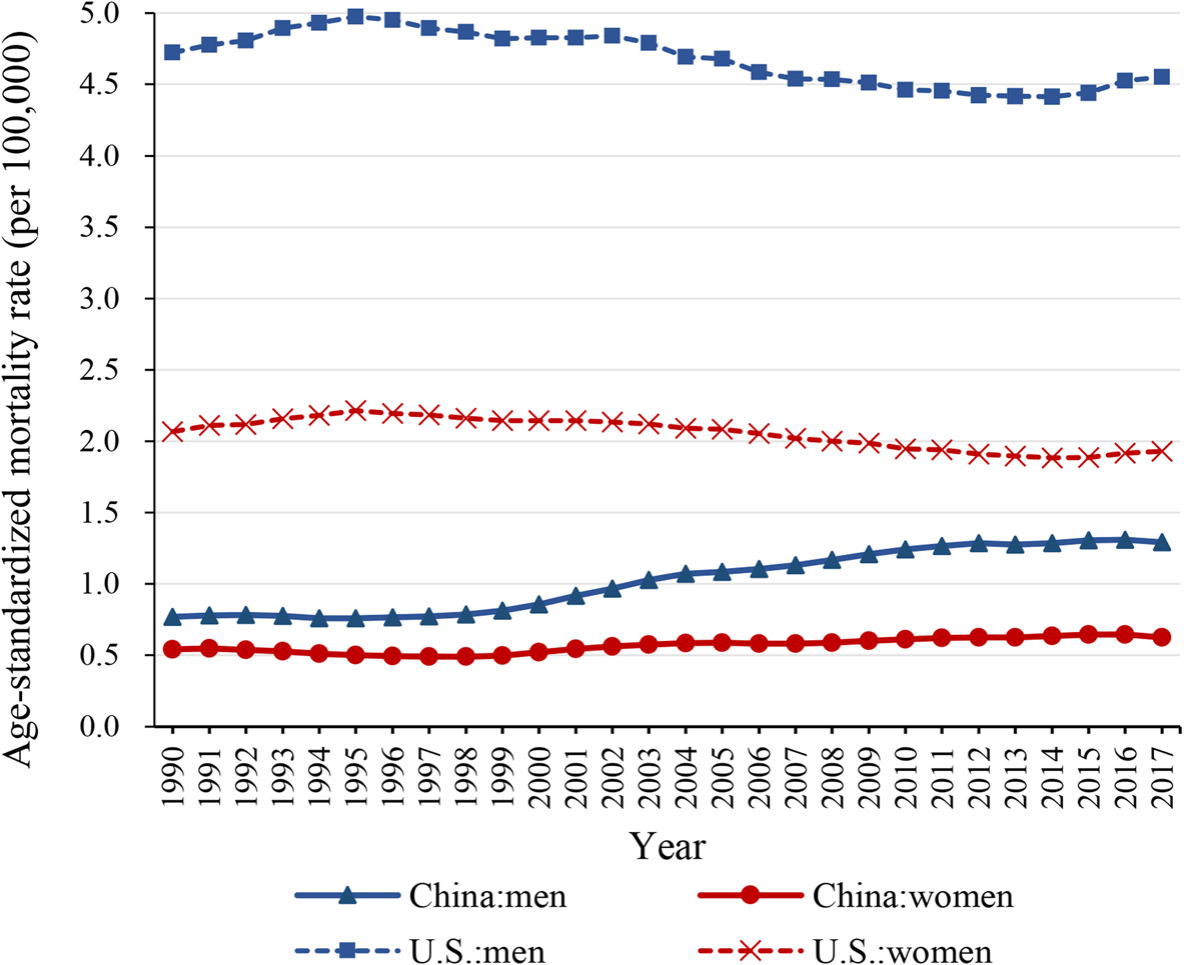
The age-standardized mortality rates of kidney cancer in China and the U.S. for both sexes at all ages, 1990–2017. Blue corresponds to men. Red corresponds to women

Figure 1 shows ASMR of kidney cancer in China and U.S. for both sexes at all ages during 1990–2017. According to GBD 2017, the ASMR of kidney cancer increased from 0.64/100,000 in 1990 to 0.94/100,000 in 2017 in China, and it decreased from 3.21/100,000 in 1990 to 3.12/100,000 in 2017 in the U.S. In 2017, the ASMRs of kidney cancer in U.S. (4.55/100,000 in men; 1.93/100,000 in women) were much higher than China (1.29/100,000 in men; 0.62/100,000 in women) for both sexes. Overall, the ASMR of kidney cancer was increasing in China for both sexes, while the ASMR in the U.S. decreased and tended to be stable during the same period. Thus, the prevalence of risk factors for kidney cancer may still impact the mortality in Chinese people.

### Trends in mortality of kidney cancer attributable to high BMI

**Fig. 2:**
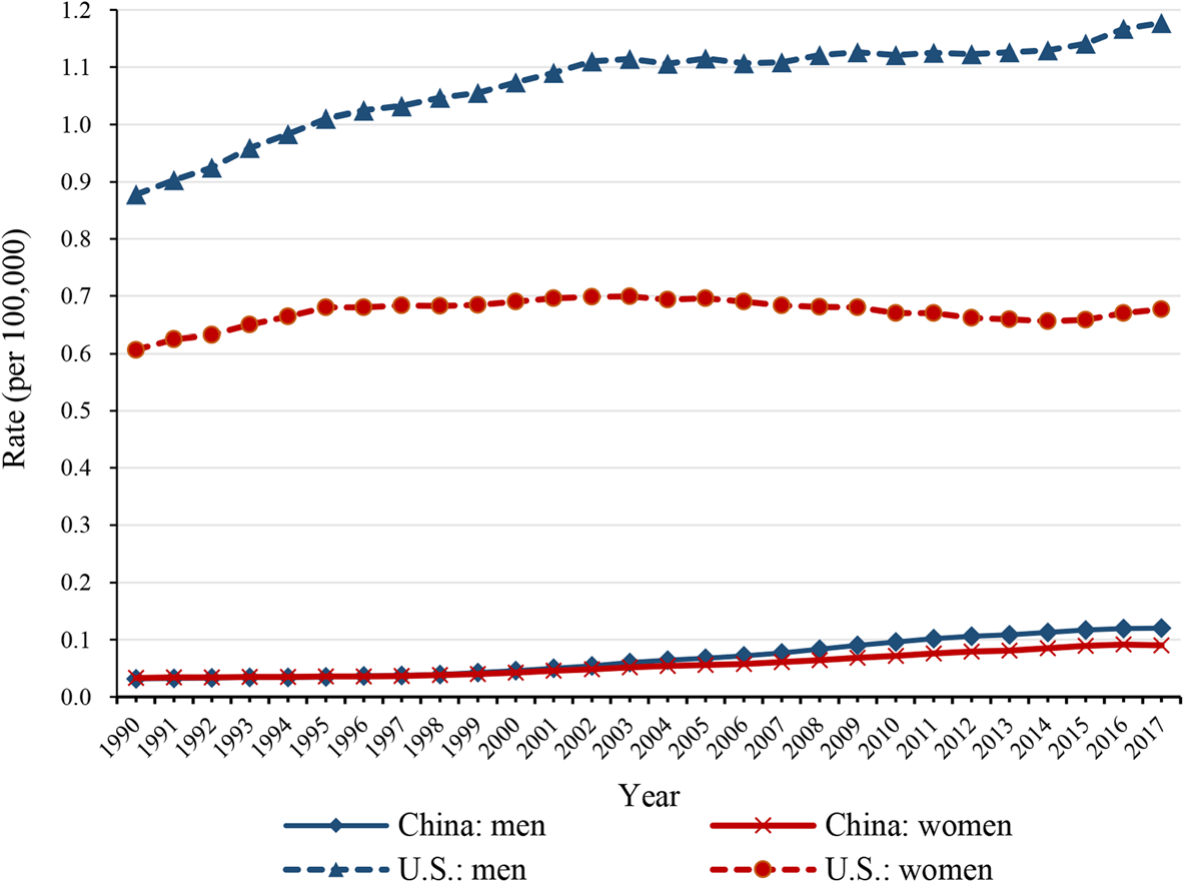
Trends in the age-standardized rates of kidney cancer mortality attributable to high BMI in China and the U.S. from 1990 to 2017, at all ages. Blue corresponds to men. Red corresponds to women

Trends in ASMR of kidney cancer attributable to high BMI from 1990 to 2017 for China and the U.S. are shown in Fig. 2. The highest rate in China was 0.12/100,000 in men and 0.09/100,000 in women in 2017. In the U.S., the highest rate was 1.18/100,000 in men and 0.70/100,000 in women during 2001–2005. China showed a low attributable mortality, compared with the U.S. In overall, a general increasing trend was observed, except for U.S. women, although it shows a low level of age-standardized mortality rate of kidney cancer attributable to high BMI in China compared with the U.S.

### The results from APC analysis of kidney cancer mortality attributable to high BMI

Age, period (year of death), and cohort (year of birth) are three independent factors that have been found to be associated with cancer mortality, and, therefore, each of these factors may affect trends in cancer mortality. We mentioned before that as we used APC model with the IE method to eliminate the non-identification problem as there is a linear relationship between the age, period and cohort. The three independent effects were presented:

### Age effect

**Fig. 3:**
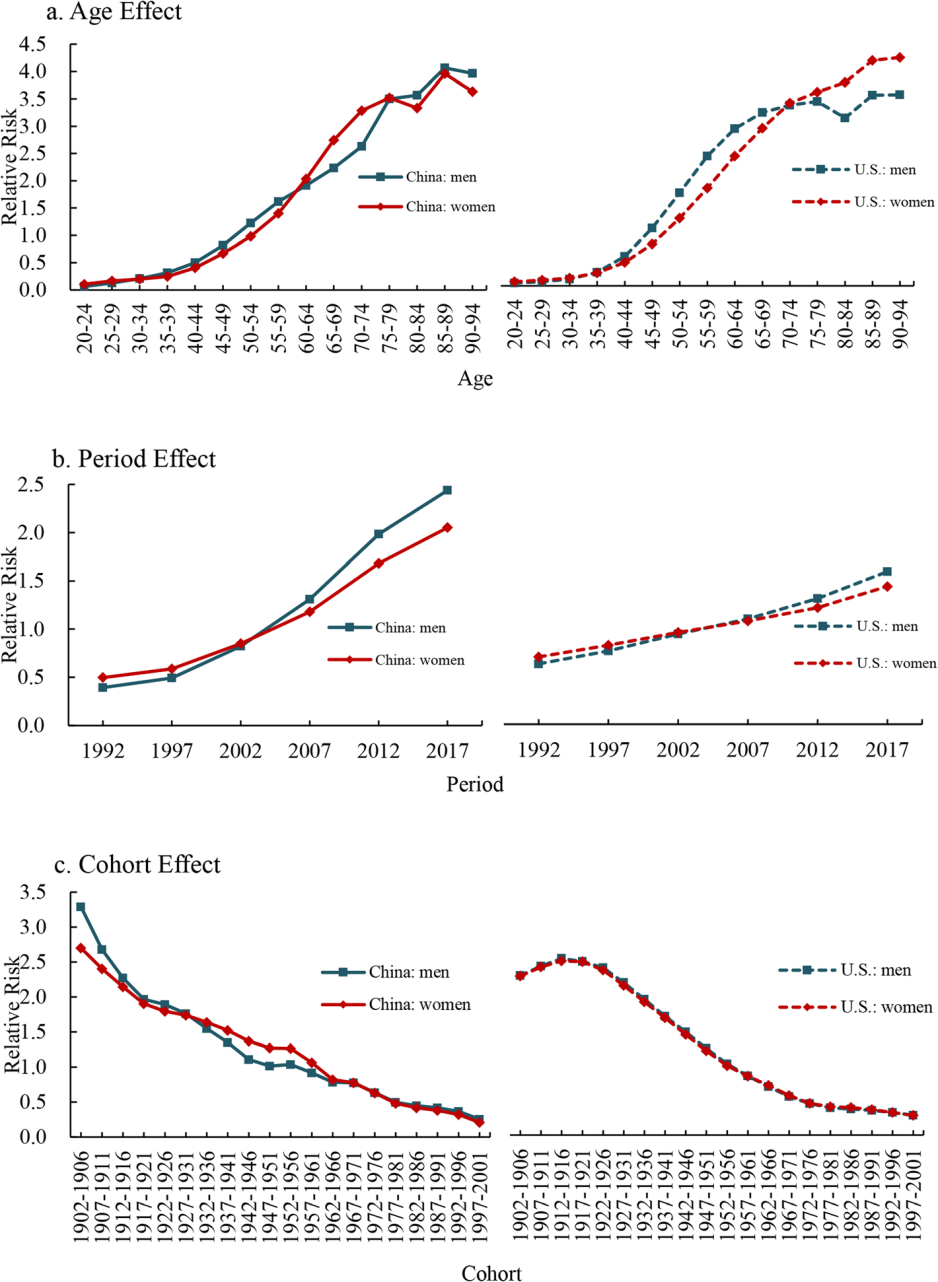
Kidney cancer mortality attributable to high BMI relative risks due to (**a**) age; (**b**) period; and (**c**) cohort effects. Blue corresponds to men. Red corresponds to women

Age effect on the mortality showed an increasing trend in both China and U.S. adults (Fig. 3a, Tables 1 and 2). The age effect indicates the RR of mortality attributable to high BMI (high-BMI-attributable mortality) varies from younger and older age groups. Generally, a similar pattern of age effect was observed between China and U.S., as well as genders. From 20 to 24 to 90–94 age group, the RR of kidney cancer mortality attributable to high BMI increased by 60.80 times and 34.17 times in men and women in China, respectively; in U.S., it increased by 69.68 times and 55.23 times in men and women, respectively (see Supplementary Table 2A and 2B).

### Period effect

The period effect on the mortality continuously increased in both China and U.S. adults, while it rapidly increased in China than U.S. during the observation period (Fig. 3b, Tables 1 and 2). This trend indicates that the RR of kidney cancer mortality attributable to high BMI increased with advancing time, and Chinese population is being more exposed to an increased risk of kidney cancer deaths. From 1992 to 2017, the RR of the mortality increased by 6.23 times and 4.14 times in men and women in China, respectively; in U.S., it increased by 2.53 times and 2.05 times in men and women, respectively (see Supplementary Table 2A and 2B). Additionally, men have a higher risk than women.

### Cohort effect

The cohort effect continuously decreased in China, and a decreasing trend was also observed in U.S. with exception of 1902–1906 and 1907–1921 birth cohort (Fig. 3c, Tables 1 and 2). From 1902–1906 to 1997–2001 birth cohort, the RR of kidney cancer mortality attributable to high BMI decreased by 92.24 and 92.29% in men and women in China, respectively; in U.S., the RR of that decreased by 87.54 and 87.64% in men and women, respectively (see Supplementary Table 2A and 2B).

## Discussion

The causes of kidney cancer are very complex, and the relevant literature shows that smoking, hypertension and obesity are the three most important risk factors [27]. According to relevant studies, the summary relative risk estimate was 1.07 (95% CI 1.05–1.09) per unit of increase in BMI [28]. With the socio-economic development, urbanization, intensified industrialization and changes in dietary and lifestyles, the exposure to kidney cancer risk factors in Chinese population has increased. From 1980 to 2000, global tobacco consumption showed an upward trend in most regions, and tobacco consumption in China increased the fastest; but decreased by 27% and 2% in the Americas and Europe [29]. According to the Chinese national nutrition and health survey, the prevalence of hypertension in the population aged over 15 years old was 5.1% in 1958–1959, 7.7% in 1979–1980, 13.6% in 1991, and 17.6% in 2002, and the age-standardized rate also showed the upward trend [15]. In 1982, overweight and obesity among the Chinese population were still very rare, at 6% and 0.6%, respectively. In 2014, the overweight rate reached 34.26% and the obesity rate was 10.98% [15]. In overall, all these factors may be related to the increased mortality of kidney cancer in this present study.

Obesity/overweight is considered to be the increased risk for kidney cancer [8, 14]. The prevalence of obesity/overweight is different between China and the U.S. Our study found that kidney cancer deaths attributable to high BMI showed an increasing tendency in China and men U.S., and the overall mortality rate of kidney cancer is still increasing in both genders in China but not in the U.S. Comparison to U.S., China has a serious attribution burden of kidney cancer death and BMI. The exposure to kidney cancer risk factors in Chinese population is increasing yearly. Potential kidney cancer risk factors include behavioural and environmental factors, comorbidities, and analgesics. Smoking, obesity, and hypertension represent established risk factors [30]. In GBD 2017 study, spatiotemporal Gaussian process regression was used to estimate risk-attributable burden and risk exposure for many risks, typically those with rich age-sex-specific data. It synthesises noisy data by borrowing strength across space, time, and age to best estimate the underlying trends for a given risk. GBD study found considerable heterogeneity across super-regions in the leading risk factors. There are also marked spatial patterns for other risks such as high BMI in central America, north Africa and the Middle East, and Oceania [22]. In our study, we obtained China and U.S. mortality data from GBD study to analyze the relationships between kidney cancer deaths and high BMI. The geographic heterogeneity in mortality rates of kidney cancer attributable to high BMI may be considerable between China and U.S.

In China, the increasing ASMR of kidney cancer attributable to high BMI was possibly associated with the prevalence of obesity [12, 13]. The increasing attributable burden and death of kidney cancer may be related to the improved diagnosis level of kidney cancer [6], and changes in exposure levels of kidney cancer risk factors in the population also have impacts on the mortality of kidney cancer [31]. Differently, a stable trend or slight decline was observed for kidney cancer mortality in U.S., which may be explained by effective improving the rising obesity trend in the US adult population over the past decades [32, 33]. In U.S., relevant researches reported that obesity prevalence remains need continue surveillance [34], and the increasing mortality attributable to high BMI was observed in men U.S. Thus, this attributable burden about BMI and kidney cancer remains need be focused.

After adjusting the three effects using the APC model, we confirmed that the differences in age-specific mortality rate patterns exist. A similar trend of age effect on kidney cancer mortality attributable to high BMI was observed between China and the U.S. adults. The age effect increased in the two areas, which indicated that the risk of the mortality of kidney cancer attributable to high BMI tended to increase in the middle-aged and younger groups. This finding may suggest that aging has driven the trend of the mortality [35, 36]. The interesting phenomenon is that age effect in the two areas was found to increase exponentially until age 70, when it continues to rise, albeit at a slower pace.

The cohort effects of the youngest and oldest age groups must be interpreted carefully because they are the small number of observations and they have larger standard errors than estimates for the middle cohorts [37]. Overall, The general trends of cohort effect showed a continuously declined trend, which indicated a decreased risk of the mortality in younger generations. Younger generations may receive good education and have a strong awareness of health [38], which may have a connection with the mortality of kidney cancer. For example, earlier birth cohorts had weak health awareness and they realized neither the occurrence of cancer nor the damage of obesity or overweight to human health. It is worth noting that cohort effect increased for 1902–1906 and 1907–1911 birth cohort in the U.S., which may indicate a higher risk in the earliest birth cohort. This finding should be treated carefully.

Here, period effects were found to be small or modest when birth cohort and age effects were both controlled. This study showed that the period effect might be the critical factor in the trend of kidney cancer mortality, because the period effect in China was significantly continuously increasing and its mortality rate was also continuously, while the period effect in U.S. slightly increased over the same period and its mortality rate tends to be stable. This relationship needs to be further verified. Increasing period effect was observed in both China and U.S. adults over the whole study period, which indicated the period risk factors might be contributed to the increase in the linear trend of the age-standardized mortality rate of kidney cancer attributable to high BMI through 1990 to 2017. However, the period trend increased rapidly in Chinese adults, compared with U.S. adults. The difference in period effect of kidney cancer was possibly related to the different risk exposure. In U.S., no significant changes in obesity prevalence in recent years [34], and some measures were conducted for improving the increasing obesity prevalence in U.S. adults [32, 33]. However, the prevalence of childhood obesity in the U.S. is rising during the past decades [39], and an increasing trend in obesity was observed for U.S. women during 2005–2014 [40]. Thus, this finding also needs to be further verified. Traditionally, obesity has been considered a problem in Western countries, while urbanisation in Asia has led to a sedentary lifestyle and overnutrition, setting the stage for the epidemic of obesity [41]. According to the 2011 China Health and Nutrition Survey, the prevalence of obesity among both Chinese adults increased significantly over the past decades, especially in men [42, 43]. Moreover, Chinese children may have a severe obesity problem. National study reported that 14.4% of children and adolescents were overweight, 11.9% were obese, and 36.8% did not meet screen-time viewing recommendations [44, 45]. The possible reason of difference between the two areas was associated to the increasing obesity prevalence. As for the different increasing period effect, the underlying reason of different extent to the trend between the two areas was possibly attributable to the fact that the increase in adult obesity in U.S. has slowed down [46]. Thus, Chinese population seems to face a more severe situation of attributable burden of kidney cancer. Our findings also may indicate inadequate measures or policies on obesity prevalence in Chinese adults, while U.S. has efforts in prevention and care of obesity, and in establishing collaborative weight management models [47, 48], and the rates of awareness, treatment and control of disease are relatively low in China population [5]. In addition, there have a higher relative risk of the mortality of kidney cancer attributable to high BMI in men than women. In China, it is more likely to be overweight/obesity in men compared with women [42, 49]. This gender difference in the relationship of kidney cancer and obesity should be further studied.

In summary, there has rapid urbanization and increasing prevalence of obesity in China, and previous study also noted a decrease in all measures of physical fitness in normal-weight adults during 2000–2014 [13], the risk of kidney cancer and high BMI need be focused. The increasing period effect indicated the period factors may be the key factor affecting the increasing mortality of kidney cancer. Thus effective measures such as promoting national fitness and low-fat dietary are needed for the prevention of obesity/overweight. It is necessary to reduce kidney cancer deaths in Chinese adults.

### Limitations

There are also some limitations in the present study. First, the data from the GBD study were supplied by the governments of the various countries and districts, which may have substantially different systems for collecting vital statistics and methods used to confirm causes of death. These factors limit the comparability of the information in the two areas. Second, despite the mortality data estimated by GBD study which incorporates methods to adjust for incomplete or missing VR and VA data, general heterogeneity in data completeness and quality, and the redistribution of so-called garbage codes (insufficiently specific or implausible cause of death codes), there might be difficult to thoroughly avoid inaccuracy of data. Therefore, our results in the present study on epidemiology of kidney cancer mortality should be treated carefully.

## Conclusions

The age-standardized morality rate of kidney cancer attributable to high BMI is rapidly increasing in China, while the mortality rate in women U.S. varies and tend to be stable in recent years. Apart from that, age effect increased and cohort effect decreased in both China and the U.S. adults, while period effect increased rapidly in China adults, compared with the U.S. adults. Obesity prevalence and China’s aging also may continuously drive kidney cancer death. Effective mearsures, such as the correct knowledge and adopting policies on body weight control and care, should be noted and conducted.

## Supplementary information

**Additional file 1: Supplementary Table 1.** Goodness-of-fit in APC models for mortality of kidney cancer attributable to high BMI. **Supplementary Table 2A.** Kidney cancer mortality attributable to high BMI estimated coefficients for the age, period and cohort effects, men. **Supplementary Table 2B.** Kidney cancer mortality attributable to high BMI estimated coefficients for the age, period and cohort effects, women.

## Data Availability

The datasets generated and/or analysed during the current study are available in the GBD repository, which was publically available at http://ghdx.healthdata.org/gbd-results-tool.

## Abbreviations

BMI: Body-mass index
APC: Age, period, and cohort
RR: Relative risk
ASMR: Age-standardized mortality rates
GBD: Global burden of disease
CDC: Center for Disease Control and Prevention
DSPs: Disease Surveillance Points
CODEm: Cause of Death Ensemble model
VR: Vital registration
VA: Verbal autopsy
ICD: International Classification of Diseases
CGLIMs: Constrained generalized linear models
