# Supplementary material for: Age-period-cohort analysis of kidney cancer deaths attributable to high body-mass index in China and U.S. adults
Source: BMC Public Health. 2020 Jun 8;20:882. doi: 10.1186/s12889-020-09007-7 (PMC7281955; doi:10.1186/s12889-020-09007-7)
Supplement: Supplementary file 1 — Additional file 1: Supplementary Table 1. Goodness-of-fit in APC models for mortality of kidney cancer attributable to high BMI. Supplementary Table 2A. Kidney cancer mortality attributable to high BMI estimated coefficients for the age, period and cohort effects, men. Supplementary Table 2B. Kidney cancer mortality attributable to high BMI estimated coefficients for the age, period and cohort effects, women. [file 12889_2020_9007_MOESM1_ESM.docx]

**Supplementary table 1** Goodness-of-fit in APC models for mortality of kidney cancer attributable to high BMI.

| **U.S. Men** | **Values** |
| --- | --- |
| Deviation | 0.10 |
| AIC | 3.32 |
| BIC | -233.89 |
| Residual df | 52 |
| **U.S. Women** |  |
| Deviation | 0.05 |
| AIC | 3.00 |
| BIC | -233.94 |
| Residual df | 52 |
| **China Men** |  |
| Deviation | 0.05 |
| AIC | 1.67 |
| BIC | -233.94 |
| Residual df | 52 |
| **China Women** |  |
| Deviation | 0.02 |
| AIC | 1.58 |
| BIC | -233.97 |
| Residual df | 52 |

AIC: Akaike Information Criterions; BIC: Bayesian Information Criterions; df: degree of freedom.

**Supplementary table 2A** Kidney cancer mortality attributable to high BMI estimated coefficients for the age, period and cohort effects, men.

| **Factor** | **China** | | | **U.S.** | | | | | |
| --- | --- | --- | --- | --- | --- | --- | --- | --- | --- |
|  | **Coefficient** | **Lower** | **Upper** |  | **Coefficient** | | **Lower** | | **Upper** |
| **Age** |  |  |  |  | |  | |  | |
| 20-24 | -2.73023 | -18.56837 | 13.10791 | -2.99167 | | -8.573872 | | 2.590533 | |
| 25-29 | -2.062868 | -11.87633 | 7.750598 | -2.606056 | | -6.671213 | | 1.459101 | |
| 30-34 | -1.563911 | -9.144944 | 6.017122 | -2.088196 | | -5.20454 | | 1.028148 | |
| 35-39 | -1.16215 | -7.450295 | 5.125994 | -1.398214 | | -3.705322 | | 0.9088935 | |
| 40-44 | -0.6882044 | -5.868247 | 4.491839 | -0.6183548 | | -2.36254 | | 1.125831 | |
| 45–49 | -0.2016355 | -4.5071 | 4.10383 | 0.0538775 | | -1.339653 | | 1.447408 | |
| 50–54 | 0.20237 | -3.457037 | 3.861777 | 0.5326371 | | -0.6132021 | | 1.678476 | |
| 55–59 | 0.4806548 | -2.644533 | 3.605843 | 0.865522 | | -0.070499 | | 1.801543 | |
| 60–64 | 0.6495988 | -1.982161 | 3.281358 | 1.057153 | | 0.3013964 | | 1.812909 | |
| 65–69 | 0.8037057 | -1.382622 | 2.990034 | 1.155649 | | 0.5452573 | | 1.76604 | |
| 70–74 | 0.9672268 | -0.8562388 | 2.790692 | 1.196766 | | 0.6727839 | | 1.720748 | |
| 75–79 | 1.253603 | -0.3110424 | 2.818249 | 1.216796 | | 0.6934117 | | 1.740179 | |
| 80–84 | 1.27198 | -0.3450859 | 2.889045 | 1.122496 | | 0.5088567 | | 1.736136 | |
| 85-89 | 1.402547 | -0.4611074 | 3.266202 | 1.249356 | | 0.4993751 | | 1.999338 | |
| 90-94 | 1.377313 | -0.9145588 | 3.669184 | 1.252239 | | 0.3254832 | | 2.178994 | |
| **Period** |  | |  |  | |  | |  | |
| 1992 | -0.9378951 | -2.922367 | 1.046577 | -0.4700769 | | -1.068711 | | 0.1285568 | |
| 1997 | -0.7101536 | -2.192651 | 0.7723439 | -0.2742066 | | -0.6799345 | | 0.1315214 | |
| 2002 | -0.1981151 | -1.261361 | 0.8651307 | -0.0662628 | | -0.3270317 | | 0.1945062 | |
| 2007 | 0.2694888 | -0.6854324 | 1.22441 | 0.0873021 | | -0.1727528 | | 0.347357 | |
| 2012 | 0.6853306 | -0.4960818 | 1.866743 | 0.2649214 | | -0.1279452 | | 0.657788 | |
| 2017 | 0.8913444 | -0.6924564 | 2.475145 | 0.4583227 | | -0.1072909 | | 1.023936 | |
| **Cohort** |  | |  |  | |  | |  | |
| 1902-1906 | 1.191451 | -3.237631 | 5.620533 | 0.8374118 | | -0.6229372 | | 2.297761 | |
| 1907-1911 | 0.9848001 | -2.544982 | 4.514582 | 0.8945002 | | -0.2797234 | | 2.068724 | |
| 1912-1916 | 0.8208844 | -2.090249 | 3.732018 | 0.9396497 | | -0.0355042 | | 1.914804 | |
| 1917-1921 | 0.6769374 | -1.752927 | 3.106802 | 0.9230338 | | 0.1030621 | | 1.743006 | |
| 1922-1926 | 0.6374805 | -1.43988 | 2.714841 | 0.8856172 | | 0.1726545 | | 1.59858 | |
| 1927-1931 | 0.5669577 | -1.337248 | 2.471163 | 0.7932151 | | 0.1263361 | | 1.460094 | |
| 1932-1936 | 0.4371207 | -1.62976 | 2.504001 | 0.6755755 | | -0.0374785 | | 1.388629 | |
| 1937-1941 | 0.3017906 | -2.090802 | 2.694384 | 0.5433363 | | -0.2746184 | | 1.361291 | |
| 1942-1946 | 0.1034758 | -2.727611 | 2.934562 | 0.4002297 | | -0.5592428 | | 1.359702 | |
| 1947-1951 | 0.0139394 | -3.312399 | 3.340278 | 0.2283594 | | -0.900894 | | 1.357613 | |
| 1952-1956 | 0.0361011 | -3.754595 | 3.826797 | 0.0312812 | | -1.286649 | | 1.349212 | |
| 1957-1961 | -0.0834802 | -4.401301 | 4.234341 | -0.1528315 | | -1.673585 | | 1.367922 | |
| 1962-1966 | -0.2445383 | -5.129349 | 4.640272 | -0.3532221 | | -2.09699 | | 1.390546 | |
| 1967-1971 | -0.2516339 | -5.643841 | 5.140573 | -0.5787745 | | -2.585009 | | 1.42746 | |
| 1972-1976 | -0.4515194 | -6.591543 | 5.688504 | -0.7867657 | | -3.129555 | | 1.556024 | |
| 1977-1981 | -0.6988809 | -8.043012 | 6.64525 | -0.9269019 | | -3.819841 | | 1.966037 | |
| 1982-1986 | -0.8024236 | -9.539433 | 7.934586 | -0.9722513 | | -4.694032 | | 2.749529 | |
| 1987-1991 | -0.8711182 | -11.69568 | 9.953439 | -1.023044 | | -6.009368 | | 3.96328 | |
| 1992-1996 | -1.002895 | -16.58687 | 14.58108 | -1.113374 | | -8.123075 | | 5.896327 | |
| 1997-2001 | -1.364449 | -35.27621 | 32.54731 | -1.245045 | | -13.90826 | | 11.41816 | |

**Supplementary table 2B** Kidney cancer mortality attributable to high BMI estimated coefficients for the age, period and cohort effects, women.

| **Factor** | **China** | | | **U.S.** | | | | | |
| --- | --- | --- | --- | --- | --- | --- | --- | --- | --- |
|  | **Coefficient** | **Lower** | **Upper** |  | **Coefficient** | | **Lower** | | **Upper** |
| **Age** |  |  |  |  | |  | |  | |
| 20-24 | -2.241005 | -16.91988 | 12.43787 | -2.581064 | | -8.000028 | | 2.8379 | |
| 25-29 | -1.790846 | -11.83837 | 8.256673 | -2.247038 | | -6.314863 | | 1.820787 | |
| 30-34 | -1.614945 | -10.38117 | 7.15128 | -1.966964 | | -5.405083 | | 1.471155 | |
| 35-39 | -1.395212 | -8.960909 | 6.170485 | -1.441826 | | -4.110705 | | 1.227054 | |
| 40-44 | -0.9064806 | -6.883777 | 5.070816 | -0.8472532 | | -2.924044 | | 1.229538 | |
| 45–49 | -0.4052484 | -5.230287 | 4.41979 | -0.2724771 | | -1.924291 | | 1.379337 | |
| 50–54 | -0.0223456 | -4.087738 | 4.043047 | 0.2111099 | | -1.131091 | | 1.55331 | |
| 55–59 | 0.3375692 | -3.049864 | 3.725003 | 0.5808366 | | -0.5104877 | | 1.672161 | |
| 60–64 | 0.7101071 | -2.023109 | 3.443324 | 0.8647378 | | -0.0123369 | | 1.741813 | |
| 65–69 | 1.009329 | -1.200022 | 3.218679 | 1.061171 | | 0.3561587 | | 1.766183 | |
| 70–74 | 1.189826 | -0.6697023 | 3.049355 | 1.208682 | | 0.6105332 | | 1.806831 | |
| 75–79 | 1.258474 | -0.4737458 | 2.990694 | 1.266613 | | 0.673663 | | 1.859564 | |
| 80–84 | 1.204567 | -0.6751591 | 3.084293 | 1.315122 | | 0.6314771 | | 1.998766 | |
| 85-89 | 1.375807 | -0.7990234 | 3.550638 | 1.417939 | | 0.580443 | | 2.255434 | |
| 90-94 | 1.290404 | -1.38657 | 3.967377 | 1.430411 | | 0.3950034 | | 2.465819 | |
| **Period** |  | |  |  | |  | |  | |
| 1992 | -0.7033937 | -2.791825 | 1.385037 | -0.3605242 | | -1.033265 | | 0.3122168 | |
| 1997 | -0.5346343 | -2.099724 | 1.030455 | -0.2016719 | | -0.6613645 | | 0.2580206 | |
| 2002 | -0.1648934 | -1.322889 | 0.9931024 | -0.0508097 | | -0.3573453 | | 0.2557259 | |
| 2007 | 0.1653357 | -0.9051042 | 1.235776 | 0.0665853 | | -0.2420675 | | 0.3752381 | |
| 2012 | 0.5193051 | -0.789077 | 1.827687 | 0.1897087 | | -0.2624865 | | 0.641904 | |
| 2017 | 0.7182807 | -1.031065 | 2.467626 | 0.3567119 | | -0.2827045 | | 0.9961282 | |
| **Cohort** |  | |  |  | |  | |  | |
| 1902-1906 | 0.9933533 | -3.88716 | 5.873866 | 0.8350981 | | -0.7469676 | | 2.417164 | |
| 1907-1911 | 0.8773029 | -2.948587 | 4.703193 | 0.890215 | | -0.3775026 | | 2.157933 | |
| 1912-1916 | 0.7642465 | -2.401377 | 3.92987 | 0.9259125 | | -0.1203968 | | 1.972222 | |
| 1917-1921 | 0.6444539 | -2.011558 | 3.300466 | 0.9209337 | | 0.0465784 | | 1.795289 | |
| 1922-1926 | 0.585818 | -1.694242 | 2.865878 | 0.871155 | | 0.112448 | | 1.629862 | |
| 1927-1931 | 0.5524358 | -1.532911 | 2.637782 | 0.7741785 | | 0.0613019 | | 1.487055 | |
| 1932-1936 | 0.4947626 | -1.760217 | 2.749742 | 0.6567246 | | -0.1199825 | | 1.433432 | |
| 1937-1941 | 0.4217425 | -2.185207 | 3.028693 | 0.529942 | | -0.3768954 | | 1.436779 | |
| 1942-1946 | 0.3140968 | -2.738869 | 3.367062 | 0.3765692 | | -0.7027951 | | 1.455934 | |
| 1947-1951 | 0.2405041 | -3.331402 | 3.81241 | 0.2003967 | | -1.080497 | | 1.48129 | |
| 1952-1956 | 0.2339507 | -3.889385 | 4.357286 | 0.0038904 | | -1.50296 | | 1.510741 | |
| 1957-1961 | 0.0620985 | -4.725004 | 4.849201 | -0.159652 | | -1.907917 | | 1.588613 | |
| 1962-1966 | -0.1965521 | -5.768838 | 5.375734 | -0.328938 | | -2.345189 | | 1.687313 | |
| 1967-1971 | -0.2505904 | -6.459633 | 5.958453 | -0.5536595 | | -2.896963 | | 1.789644 | |
| 1972-1976 | -0.4607366 | -7.590015 | 6.668542 | -0.7770676 | | -3.551869 | | 1.997733 | |
| 1977-1981 | -0.7296613 | -9.554963 | 8.09564 | -0.8860262 | | -4.290849 | | 2.518796 | |
| 1982-1986 | -0.8711379 | -11.53758 | 9.7953 | -0.9167038 | | -5.128475 | | 3.295067 | |
| 1987-1991 | -0.968247 | -13.65926 | 11.72276 | -0.9912008 | | -6.412217 | | 4.429815 | |
| 1992-1996 | -1.13837 | -18.00286 | 15.72612 | -1.116313 | | -8.412706 | | 6.18008 | |
| 1997-2001 | -1.56947 | -36.82906 | 33.69012 | -1.255455 | | -14.3054 | | 11.79449 | |
